# Epidemiology and genetic determination of measures of peripheral vascular health in the Long Life Family Study

**DOI:** 10.18632/aging.206204

**Published:** 2025-02-25

**Authors:** Deidra R. Fricke, Ryan K. Cvejkus, Emma Barinas-Mitchell, Mary F. Feitosa, Joanne M. Murabito, Sandeep Acharya, Michael R. Brent, E. Warwick Daw, Ryan L. Minster, Joseph M. Zmuda, Allison L. Kuipers

**Affiliations:** 1 Department of Epidemiology, University of Pittsburgh, Pittsburgh, PA 15260, USA; 2 Department of Genetics, Division of Statistical Genomics, Washington University School of Medicine, St. Louis, MO 63110, USA; 3 Department of Medicine, Boston University Chobanian and Avedisian School of Medicine, Boston, MA 02118, USA; 4 Section of General Internal Medicine, Boston Medical Center, Boston, MA 02119, USA; 5 Department of Human Genetics, University of Pittsburgh, Pittsburgh, PA 15260, USA; 6 Division of Computational and Data Sciences, Washington University School of Medicine, St. Louis, MO 63110, USA; 7 Department of Computer Science and Engineering, Washington University, St. Louis, MO 63130, USA; 8 Department of Medicine, Michigan State University, Grand Rapids, MI 49503, USA

**Keywords:** ankle-brachial index, peripheral arterial disease, heritability, genomewide linkage analysis, genomewide association study

## Abstract

Peripheral artery disease (PAD) is a major contributor to morbidity in older adults. We aimed to determine genetic and non-genetic determinants of PAD and ankle-brachial index (ABI) in the Long Life Family Study (LLFS). 3006 individuals had ABI assessment, including 1090 probands (mean age 89), 1554 offspring (mean age 60) and 362 spousal controls (mean age 61). Outcomes include minimum of right and left ABIs and PAD (ABI <0.9). Stepwise regression determined independent significant non-genetic correlates of ABI and PAD. Genomewide association and linkage analyses were adjusted for age, sex, study center, significant principal components, and independent predictors. All analyses accounted for familial relatedness. Median ABI was 1.16 and 7.4% had PAD (18.2% probands, 1.0% offspring, 1.9% controls). Correlates of PAD and lower ABI included age, SBP, and creatinine (ABI only); BMI (ABI only), HDL (ABI only) and DBP (PAD only); and antihypertensive use, current smoking, female sex (ABI only), and high school noncompletion (ABI only). Genomewide linkage identified 1 region (15q12-q13) and association identified 3 single nucleotide polymorphisms (rs780213, rs12512857, rs79644420) of interest. In these families, PAD prevalence was low compared to other studies of older adults. We identified four genomic sites that may harbor variants associated with protection from PAD.

## INTRODUCTION

Peripheral artery disease (PAD) is underdiagnosed yet highly prevalent within the United States and internationally, with an estimated 8.5 million Americans living with known or unknown PAD [1]. Peripheral artery disease is the progressive stenosis and/or occlusion of arteries not involving the heart or brain. It generally refers to disease occurring in arteries within the lower extremities, and it can lead to claudication, cramping, and recurrent fatigue [2]. In addition to its own symptoms, PAD is associated with an increased risk of coronary artery disease and predicts future cardiac events and stroke [3]. Given that it is estimated that over half of all PAD patients are asymptomatic, the condition is thought to be widely underdiagnosed, though even if asymptomatic, the risk of future cardiovascular disease (CVD) remains [1]. Therefore, it is important to better understand the risk factors and genetic variants to potentially improve prediction and outcomes related to PAD.

The presence of PAD in the lower extremities can be assessed by measurement of the ankle-brachial index (ABI) [4], which is a measurement of the systolic blood pressure (SBP) in the posterior tibial artery (ankle) divided by the SBP in the brachial artery. Typically, a normal ABI = 1.00–1.40, borderline ABI = 0.90–0.99, and abnormal is ABI <0.9, with severe disease and an increased risk of major limb amputation at ABI ≤0.4 [2, 3]. Conversely, an ABI above 1.4 is indicative of a non-compressible or an extremely stiff and calcified tibial artery, which is more closely related to stroke and heart failure than the atherosclerotic disease [3]. One of the most notable risk factors for PAD or lower ABI is increased age, with global estimates suggesting the prevalence of PAD is ~5% among individuals aged 40–44 years, which increases to ~12% by age 70–74 years [1]. However, there is a dearth of data on ABI and PAD prevalence from the general population in older adults. This is particularly problematic when assessing the burden of disease, considering it is so often asymptomatic and underdiagnosed. Other known risk factors include traditional CVD risk factors, such as hypertension, diabetes, kidney disease, smoking, hyperlipidemia, and lower socio-economic status [1, 5].

ABI and PAD are also partially determined by genetic variation. Common variants in 17 loci have been associated with ABI and/or PAD in different populations such as Japanese [5], Hispanic Americans [6], Veterans [7], and via electronic health records [8]. A meta-analysis in 2012 found variants in the 9p21 locus to be of particular importance [9], though the underlying function of these variants is still unknown. The candidate gene association resource (CARe) consortium, which included a ~50 k candidate SNP panel, also identified 3 risk-factor related genes associated with ABI and PAD (*SYTL3:* lipoprotein(a),* TCF7L2k:* diabetes, and* CYUP2B6:* smoking), though none replicated in external studies [10]. Additionally, one genomewide linkage study of ABI, conducted in African Americans and non-Hispanic Whites [11], identified loci on 1p, 3p, 3q, 6q, 7q, 10p, and 16p. However, outside of the 9p21 locus, there have been no genomic loci with strong effects on ABI or PAD, suggesting there may still be novel variants or regions of the genome underlying ABI and/or PAD yet to be identified.

Therefore, we characterized ABI and PAD in 3006 individuals from the Long Life Family Study (LLFS), a multi-center study of exceptionally long-lived individuals across three U.S. field centers. We also determined the independent correlates of ABI and PAD, including demographic, lifestyle, and medical factors. Lastly, we identified genetic determinants of ABI and PAD using both genomewide association (GWAS) and genomewide linkage approaches. This work aimed to comprehensively assess the correlates of ABI and PAD in long-lived individuals, who we hypothesize will harbor novel genetic determinants underlying peripheral vascular health.

## RESULTS

LLFS participants were 70 years old on average, and 56% were female (Table 1), with probands being 89 years old on average and offspring around 60 years old on average. Participants were very likely to have completed a high school education (92%), remained physically active (67% walk ≥3 hours/week), and were unlikely to be current smokers (5%). About half of the participants were hypertensive, and about 9% diabetic; however, moderate dyslipidemia and/or use of a statin was very common (90%) and driven by LDL-cholesterol >100 and/or use of a statin (33%). Overall, LLFS participants differed by generation such that the ~28-year younger on average offspring generation had a lower prevalence of hypertension, diabetes and medication usage, and had better kidney function and greater educational attainment. However, they also drank more alcohol per week, were more likely to be a current smoker, had higher BMI, and greater serum lipoprotein cholesterol measures than the proband generation (all *P* < 0.05).

**Table 1:** Long Life Family Study characteristics.

	**Overall (*N* = 3006)**	**Probands (*n* = 1090)**	**Offspring and spousal Controls (*n* = 1916)**	**Sex-adjusted *P*-value**
Age (years)	70.6 ± 15.8	88.7 ± 6.9	60.3 ± 8.3	<0.0001
Female sex (%)	56.2	53.2	57.9	<0.0001^a^
Field center (%)				<0.0001
Pittsburgh	35.7	32.7	37.5	
Boston	36.1	30.6	39.1	
New York	28.2	36.7	23.4	
BMI (kg/m^2^)	27.3 ± 5.0	26.2 ± 4.2	28.0 ± 5.3	<0.0001
Current smoker (%)	4.7	1.1	6.8	<0.0001
Walk ≥3 hrs/week (%)	67.4	66.8	67.7	0.9660^b^
>7 alcoholic drinks/week (%)	12.3	7.0	15.4	<0.0001
Completed high school (%)	92.4	82.5	98.1	<0.0001
Hypertension (%)	52.2	68.6	42.8	<0.0001
SBP (mmHg)	130.3 ± 21.5	138.6 ± 24.3	125.6 ± 18.1	<0.0001
DBP (mmHg)	76.2 ± 10.9	73.4 ± 11.3	77.7 ± 10.2	<0.0001
Hypertension medication (%)	46.9	69.6	33.9	<0.0001
Diabetes (%)	9.3	11.7	7.9	0.0011
Fasting glucose (mg/dL)	93.7 ± 20.6	95.6 ± 20.0	92.7 ± 20.9	0.0011
HbA1c	5.65 ± 0.58	5.77 ± 0.56	5.58 ± 0.58	<0.0001
Diabetes medication (%)	5.7	6.7	5.2	0.1410^b^
Dyslipidemia (%)	88.9	87.8	89.8	0.0408
Fasting LDL (mg/dL)	114.2 ± 33.6	108.6 ± 34.2	117.3 ± 32.9	<0.0001
Fasting HDL (mg/dL)	57.8 ± 17.1	55.4 ± 15.7	59.1 ± 17.8	<0.0001
Fasting triglycerides (mg/dL)	113.5 ± 71.1	110.1 ± 55.2	115.4 ± 78.6	0.0374
Lipid-lowering medication (%)	32.7	37.1	30.2	0.0003
Serum creatinine (mg/dL)	1.0 ± 0.3	1.2 ± 0.4	0.98 ± 0.2	<0.0001
Nitroglycerine (%)	26.7	42.8	17.5	<0.0001

^a^Differences in sex by generation are shown without adjustment for sex. ^b^All variables, with the exception of the percentage of those taking diabetes medication and the percentage of individuals who walked three or more hours per week, were statistically significantly different between the proband and offspring generations.

In all LLFS, the median ABI was 1.16 and a PAD prevalence of 7.4% (Supplementary Table 1). These differed significantly by generation, with probands having lower ABI than offspring (1.10 vs. 1.19, respectively) and greater PAD prevalence (18.2 vs. 1.0, respectively). The median ABI in the spousal control group was 1.20. When compared to their spouses and adjusting for age, sex, and field centers, offspring of long-lived families had statistically similar ABI and PAD prevalence (both *P* = 0.5). However, PAD prevalence was almost 2 times greater in spouses compared to the offspring of long-lived parents (1.9 vs. 1.0). The mean age (range) of the offspring generation was 60.1 (30–87) and for spousal controls, it was 60.9 (24–83) years (*P* for difference = 0.07). Offspring were 60.1% female, while spousal controls were 48.3% female (*P* for difference < 0.0001).

Significant independent predictors of lower (or worse) ABI and prevalent PAD are shown in Table 2. After forcing age, sex, and field centers into all models, independent correlates of lower ABI included increased age, SBP, and serum creatinine; decreased BMI and HDL-cholesterol; and female sex, current smoking, not completing high school, and hypertensive medication. For prevalent PAD, after forcing age, sex, and field centers into all models, independent correlates of PAD prevalence included increased age and SBP, decreased DBP, current smoking, and hypertensive medication. In these fully adjusted models, residual genetic heritability of peripheral vascular disease measures in the LLFS was moderate at 0.115 for ABI and 0.233 for PAD.

**Table 2:** Stepwise-selected independent predictors of peripheral vascular health measures in the LLFS.

**Predictor**	**Unit 1-SD^a^**	**Lower ABI^b^**	**Prevalent PAD (ABI <0.9)**
Age (years)	15.7	11% (*P* < 0.0001)	4.44 (3.49, 5.65)
Female Sex	1	15% (*P* < 0.0001)	0.89 (0.64, 1.23)
BMI (kg/m^2^)	5.0	−3% (*P* < 0.0001)	–
Current smoker	1	11% (*P* < 0.0001)	2.91 (1.07, 7.89)
Completed high school	1	−6% (*P* = 0.0280)	–
SBP (mmHg)	21.5	5% (*P* < 0.0001)	1.58 (1.32, 1.88)
DBP (mmHg)	10.9	–	0.68 (0.55, 0.84)
Hypertension medication	1	4% (*P* = 0.0010)	1.56 (1.09, 2.25)
Fasting HDL (mg/dL)	17.1	−2% (*P* = 0.0154)	–
Serum creatinine (mg/dL)	0.3	2% (*P* = 0.0009)	–
H_r_^2^, *P*-value^c^	–	0.115, *P* = 0.0009	0.233, *P* = 0.0660

^a^Effects are per 1-SD (e.g., unit) difference in each predictor and shown as: percent mean difference (*P*-value) for ABI and Odds Ratio (95% CI) for PAD. Age, sex, and field centers were forced into all models. ^b^A lower ABI indicates worse peripheral vascular health, and positive percent mean differences in this column represent reductions in ABI (on average) per 1-SD increase in the relevant predictor. ^c^Residual genetic heritability estimates include adjustment for all covariates listed above plus field centers.

**Figure 1:**
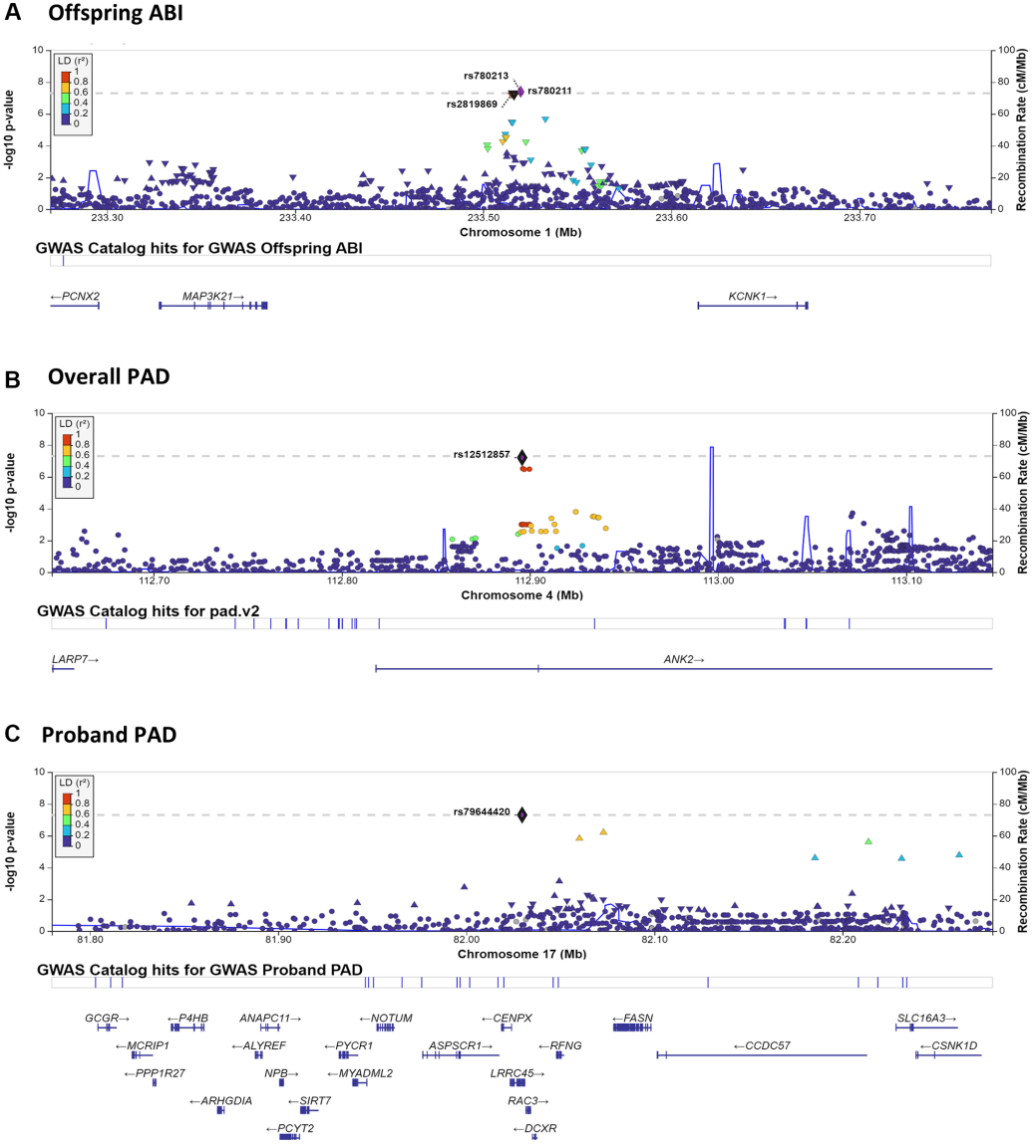
**LocusZoom plots surrounding notable GWAS results.** LocusZoom plots are shown for each of the three significant or highly suggestive GWAS results shown in Table 3. Plots include 250 KB up- and down-stream of the index SNP. Results by panel include: (**A**) chromosome 1– Offspring ABI; (**B**) chromosome 4– Overall PAD; (**C**) chromosome 17– Proband PAD.

**Figure 2:**
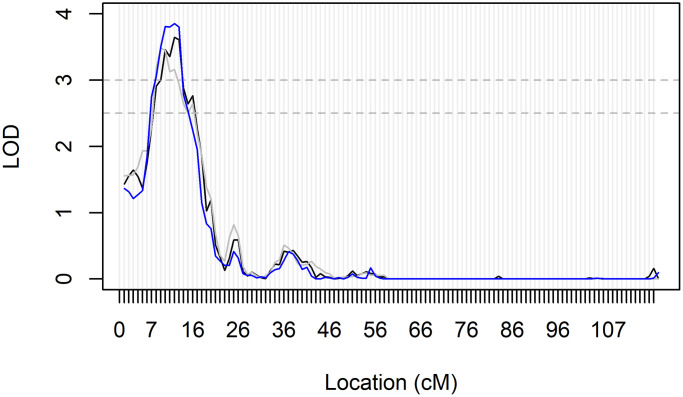
**Genomic region within the significant linkage peak for ABI in All LLFS participants.** Fully adjusted LOD-scores on chromosome 15 from all three bins of mIBD estimates for linkage analyses of ABI in all participants. Significance and suggestive evidence of linkage are indicated by the horizontal dashed lines at LOD = 3.0 and LOD = 2.5, respectively. The peak reached its maximum LOD score at 11cM, and had a range of 6–16cM. This region maps to 26,267kb-31,213 kb on chromosome 15 and the 26 genes are located within this genomic region. They include *GABRB3, GABRA5, GABRG3, OCA2, HERC2, GOLGA6L24, GOLGA8F, GOLGA8G, GOLGA6L25, GOLGA8M, GOLGA6L7, APBA2, ENTREP2, NSMCE3, TJP1, GOLGA8J, GOLGA8T, CHRFAM7A, GOLGA8R, GOLGA8Q, GOLGA8H, ARHGAP11B, ENSG00000284906, FAN1, MTMR10,* and *TRPM1* (as generated from the UCSC genome browser; GRCh38/hg38 accessed on August 14, 2023)*.*

Genomewide association and linkage analyses identified four genomic regions with significant or highly suggestive evidence of genetic variability related to ABI and PAD (Table 3, Figures 1, 2 and Supplementary Figure 1). No genomic inflation was detected as assessed by Q-Q plots or the genomic inflation factor (all λ <1.04; Supplementary Figure 2). Due to the very large age range of the LLFS and the strong age effect on ABI and PAD prevalence, we tested genomewide data in both the overall LLFS and stratified by generation (Supplementary Figures 1–3). In the full LLFS, we found significant evidence for linkage to ABI on chromosome 15 between 6–16cM (peak LOD = 3.85 11cM; Figure 2). We also found strongly suggestive evidence of association with PAD on chromosome 4 at rs12512857 (*P* = 6.3 × 10^−8^; Figure 1B and Supplementary Figure 1A right). In generation-stratified analyses, we found significant evidence of association with PAD in the proband generation on chromosome 17 at rs79644420 (*P* = 5.0 × 10^−8^; Figure 1C and Supplementary Figure 1B right). We also found significant evidence of association with ABI in the offspring generation on chromosome 1 at rs780213 (*P* = 4.0 × 10^−8^; Figure 1A and Supplementary Figure 1C). No significant SNPs from the LLFS GWAS showed suggestive or significant association to ABI or PAD using the same adjustments in the Framingham Heart Study (data not shown).

**Table 3:** Genomewide significant and highly suggestive associations with peripheral vascular health measures in the LLFS.

**Measure and analysis^a^**	**Maximum significance estimate**	**CHR**	**Index SNP (other sig SNPs) or Peak (range)**	**MAF**	**β ± SE**	**Closest gene and location (other nearby genes)**
**Overall (*N* = 3006)**						
ABI GWAS	None	–	–	–	–	–
ABI Linkage	LOD = 3.85	15	11cM (6-16cM)	–	–	26 genes in ~4.9 mb region
PAD GWAS	*P* = 6.3 × 10^−8^	4	rs12512857	0.021	2.28 ± 0.42	*ANK2* intronic (*LARP7*)
PAD Linkage	None	–	–	–	–	–
**Proband generation (*N* = 1090)^b^**						
ABI GWAS	None	–	–	–	–	–
ABI Linkage	None	–	–	–	–	–
PAD GWAS	*P* = 5.0 × 10^−8^	17	rs79644420	0.015	3.43 ± 0.63	*LRRC45* intronic (*RAC3, DCXR, CENPX, RFNG, ASPSCR1, FASN*)
PAD Linkage	None	–	–	–	–	–
**Offspring generation and spousal controls (*N* = 1916)^b^**						
ABI GWAS	*P* = 4.0 × 10^−8^	1	rs780213 (rs780211, rs2819869)	0.210	−0.09 ± 0.02	*KCNK1* 3’ upstream (*MP3K21, PCNX2*)
ABI Linkage	None	–	–	–	–	–

^a^All results are shown for the fully adjusted models based on the independent correlates from Table 2 and use methodology to account for family structure, as well as, inclusion of significant PCs to control for population sub-structure. Adjustments for ABI include: age, sex, field centers, significant PCs, systolic blood pressure, body mass index, completion of a high school education, antihypertensive medication use, smoking status, high-density lipoprotein, and creatinine level. Adjustments for PAD include: age, sex, field centers, significant PCs, smoking status, systolic blood pressure, diastolic blood pressure, and antihypertensive medication usage. ^b^PAD stratification was limited to the proband generation due to low PAD prevalence in offspring.

## DISCUSSION

We provide a detailed analysis of the correlates of ABI and PAD in older adults in the LLFS, including genetic effects. PAD was present in 7.4% of the study overall, with a drastic difference by generation reflecting the well-known strong effect of age on disease presentation (18% in probands, 1% in offspring). While not statistically significant, perhaps due to the low prevalence and power, PAD appeared slightly less common in offspring from long-lived families than in their spouses, suggesting a potential protective effect in these families (1% vs. 2%, *P* = 0.5). Both PAD and lower ABI were independently associated with greater age, systolic BP, smoking, and hypertensive medication. ABI was also lower in females, those with lower BMI and less education, and those with higher HDL and creatinine. PAD prevalence was also associated with lower diastolic BP. In genetic analyses, we identified three loci of interest (2 for PAD; 1 for ABI) from GWAS analyses; an additional genomic region of interest was identified for ABI through genomewide linkage analyses. Overall, these analyses helped to explore genetic determinants of peripheral vascular health of the LLFS, which is a uniquely long-lived group of individuals.

Compared to previous studies of ABI and PAD prevalence in older adults, LLFS appears to have lower prevalence of disease. NHANES data estimate that the prevalence of PAD among individuals aged 40–44 is 5%, and among people aged 70–74, it is 12% [1]. In LLFS family members aged 70–74, PAD prevalence was only 4.4%. Another systematic review of global PAD prevalence in high-income countries showed PAD prevalence of ~18% at age 85–89 versus ~9% at age 60–64 years [12]. The LLFS proband generation had a PAD prevalence of 18% (average age 90 years), but the offspring generation only had a prevalence of 1.0% (average age 60 years). Therefore, individuals from long-lived families have a lower overall prevalence of PAD compared to peers of the same age both globally and in the US, and this difference is most striking in younger adults. This trend was also observed within the LLFS comparing offspring to their spouses. Therefore, there are likely some protective effects, perhaps environmental or biological, regarding peripheral vascular health in the LLFS.

The independent predictors of ABI and PAD in the LLFS were very similar to predictors identified in other previous studies [1, 13–15]. They included age, hypertension, smoking, kidney disease, low HDL and lower SES (proxied by educational attainment in the LLFS). A notable exception is that previous studies in other cohorts regularly identify hyperlipidemia [15], and diabetes [14] as strong predictors of ABI and/or PAD. However, these were not significant predictors in the LLFS in multivariable models. Low HDL was a significant predictor of lower ABI in LLFS, but the typical elevated total or LDL- cholesterols [15] were not. While moderate dyslipidemia is very common in LLFS, it is driven by only slightly elevated LDL-cholesterol. This suggests that the general lipoprotein profile of LLFS participants is relatively healthy compared to the general population even if not considered clinically ideal; therefore, elevated cholesterol is not likely to be a strong driver of ABI and PAD in the LLFS. Similarly, diabetes prevalence is quite low in LLFS due to the criteria for entering the study, and is thus possibly underpowered to show a significant association with ABI or PAD in LLFS participants. Hyperlipidemia and diabetes are not independent predictors of ABI and PAD in the LLFS compared to previous studies, but this is largely reflective of the generally ‘healthier’ LLFS compared to other cohorts.

We identified four genomic regions with association or linkage to ABI and PAD after adjustment for covariates (on chromosomes 1, 4, 15, and 17). Of note, the commonly reported 9p21 locus [9] did not show evidence of association with ABI or PAD in our analyses. However, rs6475897, which maps to the 9p21 locus, did show suggestive evidence of association for ABI in the offspring generation alone (MAF: 0.096, *P* = 6.3 × 10^−6^). No other previous genomic analysis results [5–8, 10, 11] were replicated in the current analysis. Therefore, these 4 genomic signals appear to be novel and may harbor variation unique to the long-lived families of the LLFS.

There are three genes (*KCNK1*, *MAP3K21*, and *PCNX2*) located near the three SNPs (rs780213, rs780211, rs2819869) that were identified on chromosome 1q42 in the GWAS of ABI in the offspring generation. While rs780213 was genomewide significant in the stratified analyses of offspring only, the magnitude and direction of effect were similar in probands, suggesting that there is not a strong effect of age or interaction, but rather more power in the larger offspring group (β_off_ (*P*-value_off_): −0.09 (4.0 × 10^−8^) vs. β_pro_ (*P*-value_pro_): −0.06 (0.042), proband data not shown). *KCNK1* encodes a potassium channel with somewhat poorly-defined physiologic roles in both the heart and the brain [16] and has been identified as a potential prognostic biomarker and therapeutic target in breast cancer [17]. Both *MAP3K21* and *PCNX2* have no previous evidence of an effect in the vasculature, but have an implicated role in cancers, such as breast cancer metastasis [18], glioblastomas [19] (*MAP3K21*), and thyroid cancer (*PCNX2*) [20]. Therefore, *KCNK1* is the most likely candidate gene, but additional research would be needed to identify the causal gene and variant(s) in the LLFS.

The region surrounding the SNP (rs12512857) that is suggestively associated with PAD in the overall study GWAS contains two genes: *ANK2* and *LARP7*. *LARP7* encodes a protein component of the 7SK small nuclear ribonucleoprotein, which inhibits the cyclin-dependent kinase required for RNA polymerase II to begin elongation [21]. This gene has been implicated in the loss of arterial elasticity and gain of vascular stiffness in mouse models [21] and is downregulated in humans with heart failure [22]. *ANK2* encodes ankyrin-B, which interacts with ion channels and transporters and has been associated with congenital long QT arrhythmia syndrome, episodes of atrial fibrillation, and other types of cardiac dysfunction [23, 24]. In mouse models, haploinsufficiency can lead to altered calcium handling within the heart and severe cardiac phenotypes, including sudden death [25]. However, no specific vascular phenotype was noted in these studies, and cardiac function is generally distinct from the pathogenesis of PAD. Given the noted function in maintaining arterial elasticity, a vascular pathology that can contribute to decreased ABI and incidence of PAD, *LARP7* may be the most likely candidate gene in this genomic locus.

The significant linkage peak on chromosome 15q12-q13 for ABI (max LOD = 3.85) spanned 26,267kb-31,213 kb on chromosome 15, a region that contains 26 genes (Figure 2). Of the 26, 11 genes are members of the Golgin subfamily (gene names: *GOLGA##*), a set of coiled-coil proteins localized to the Golgi apparatus [26], with no known effects on vascular disease or physiology. Three other genes are subunits of a chloride channel receptor for gamma-aminobutyric acid, a neurotransmitter in the nervous system that has been indicated in conditions involving epilepsy [27]. The remaining 11 genes in the region encode proteins associated with neurologic disorders (*APBA2, CHRFAM7A, MTMR10, FAN1*), skin and eye pigmentation or development (*OCA2, HERC2, TJP1, TRPM1*), nephritis (*ARHGAP11B, MTMR10, FAN1*), and lung disease (*ENTREP2, NSMCE3*). *ENSG00000284906* encodes a novel protein with no known function. None of these genes appear to be particularly strong candidates for association with ABI. However, there are multiple GWAS associations in this large genomic region for other relevant traits, including triglyceride levels (*n* = 4 studies), longevity (*n* = 2 studies), SBP (*N* = 2 studies), and coronary artery calcification (two studies; as per the GWAS Catalog: ebi.ac.uk/g was accessed 08/14/2023). Therefore, this region identified through genomewide linkage analysis in exceptionally long-lived families may harbor novel genomic variants for ABI.

Lastly, there were numerous genes on chromosome 17q25 in the area surrounding the genome wide significant SNP (rs79644420) in the proband generation for PAD (Figure 1C). Being heterozygous for this relatively uncommon SNP in LLFS (MAF = 0.015) was associated with greater PAD prevalence, particularly in the proband generation (72% in GA vs. 18% in AA in probands; 22% in GA vs. 7% in AA overall). However, the effect was opposite in offspring, perhaps suggesting a modification by aging (0% PAD in GA vs. 1.2% PAD in AA), though numbers were extremely low and may be unreliable. A previous study examining traffic-related air pollution and PAD [28] found significant associations between SNPs in the *SLC16A3* and *CCDC57* genes, which are ~200 kb downstream, but in moderate LD with the lead SNP in our study (Figure 1C). Additionally, *SIRT7* (~100 kb upstream of our lead SNP) may be associated with Monckeberg arteriosclerosis [29], which is a form of arterial hardening characterized by extensive medial calcification [30]. As medial calcification plays a strong role in PAD pathogenesis [31], *SIRT7* may also be a strong candidate gene. Lastly, given the relatively large number of nearby genes (*N* = 23), we tested mRNA gene expression from peripheral blood RNAseq in the LLFS with ABI and PAD adjusting for the full model covariates plus blood cell counts. Of the 23 genes, 2 were expressed in the peripheral blood, *CSNK1D* and* PCYT2,* and greater expression of these genes showed suggestive association with PAD (*P* = 0.015 and *P* = 0.013, respectively). *PCYT2* is a strong candidate gene for aging, in general, as it has been shown to be essential for skeletal muscle function [32] and the decline in aging muscle of humans [33]. While there is no clear link established to ABI or vascular disease in the known function of *PCYT2*, this LLFS-specific gene expression association plus the differential effect of rs79644420 by generation (e.g., age group) makes it a strong candidate gene for these analyses that should be further evaluated in LLFS and other vascular aging research settings.

This study has several strengths, including its family design, which allows for both GWAS and linkage approaches, and the extensive health history and phenotype data available, which allowed for thorough identification of and adjustment for potentially confounding effects. Low ABI, or PAD, is a condition that commonly affects older individuals, who are typically susceptible to many other chronic conditions and cardiovascular diseases. Therefore, studying a healthier than average population can reduce some of the confounding effects from other comorbidities and poor lifestyle habits that are otherwise present in the general population. This unique study design of long-lived individuals also allows for the identification of potentially protective effects. However, this may also suggest that findings from the LLFS may not be generalizable to the broader population. One other limitation is that these analyses were not sufficiently powered to examine non-compressible arteries. Despite these limitations, the unique characteristics of the LLFS facilitated the identification of novel genetic regions of interest for ABI and PAD.

In conclusion, we have shown that individuals from long-lived families appear to have healthier peripheral arteries, as characterized by a lower prevalence of PAD, than their similar aged peers. However, the general correlates of ABI and PAD are similar to those found in other studies. Genetic analyses identified four genomic regions that may harbor variants associated with ABI and/or PAD in the LLFS. At least three of these four regions were novel, with the fourth being only nearby but not identical to a previous GWAS signal for PAD. A number of candidate genes identified have a relationship to vascular stiffening, but none have been implicated in atherosclerosis. While PAD is generally considered to be reflective of atherosclerotic deposition, these findings may suggest that protection from vascular stiffening drives the lower prevalence of PAD in the LLFS. Future work to further refine these genomic regions within the LLFS to identify potential causal genes or variants is needed. Notably, the findings from this work may provide novel insight into the underlying mechanisms of PAD and, perhaps, have prevention or therapeutic impacts.

## MATERIALS AND METHODS

### Long Life Family Study

The Long Life Family Study is a family-based cohort study of exceptional longevity that recruited families at four study centers (Boston, New York, Pittsburgh, and Denmark) based on being exceptionally long-lived (aged 80+ years in the US), having one or more siblings who were also exceptionally long-lived, and having at least one offspring. The study recruited as many siblings, spouses, and offspring of the long-lived individuals as possible. Interested offspring spouses served as non-long-lived familial controls. Other characteristics of family eligibility, recruitment, and composition have been previously described [34, 35]. In total, the LLFS recruited 4559 men and women from 2006 to 2009; however, only participants in the US underwent ABI measurement (*N* = 3086). We have complete data on ABI, genetic markers, and covariates of interest in 3006 individuals who serve as the basis of the current analysis. Written informed consent was obtained from each LLFS participant using forms and procedures approved by each participating institution’s Institutional Review Board.

### Ankle-brachial index measurement

During their in-home LLFS visit, participants underwent ABI measurement using a handheld 8-megahertz Doppler probe and an Omron HBP-1300 digital blood pressure machine by centrally trained and certified research assistants. Prior to study certification and the collection of ABI data, all research assistants were required to collect ten simultaneous readings of age-eligible participants that were within ± 4 mmHg of another staff member and perform the exam according to a quality control checklist. Systolic blood pressure (SBP) was taken twice at the brachial, left posterior tibial, and right posterior tibial arteries while participants were lying flat or in a semi-recumbent position. The right arm was used for brachial SBP measurement unless the SBP in the left arm was >10 mmHg greater than that in the right (*N* = 287), or if there was a prohibitive medical condition in the right arm (*N* = 57). If a posterior tibial SBP was not detectable, the dorsalis pedis SBP was used (*N* = 203).

The ABI ratio was calculated by taking the average of each arterial location SBP. Then, the mean right and left ABI was calculated for each side using the right or left leg SBP measurement divided by the mean brachial SBP, respectively. The minimum of the right and left ABI was used in the analysis. PAD was defined as any participant with a minimum ABI <0.9. Any individual with both sides missing an ABI measurement (e.g., non-compressible artery) and/or with a minimum ABI >1.4 were excluded from analyses as these values have also been associated with increased cardiovascular risk [3]. In total, 285 individuals were excluded for these two reasons, and of these, only 173 were designated as having non-compressible arteries. Furthermore, only 28 of these individuals were in the offspring generation. Therefore, this analysis is not sufficiently powered to examine the genetic determinants of non-compressible arteries. If only one side was missing and/or had an ABI >1.4, then the other side’s value was used. In total, ABI data were available for 3,006 participants.

### Other data collection

In addition to the ABI measurement, participants underwent standard clinical exams and interviewer-administered questionnaires regarding demographics, health history, and lifestyle habits. Smoking was defined as current versus past or never. Data on physical activity was based on walking as the predominant form of exercise. Participants were divided into those who walked 3 or more hours per week on average as being physically active, versus those who did not. Alcohol use was categorized into more than 7 drinks per week on average versus less. Self-report completion of a high school education was used as a marker of general SES. All current medications were shown to the study staff, who then noted the name, dosage, form, and duration of use in the home. These were later categorized into therapeutic subgroups according to the anatomical therapeutic chemical (ATC) classification system for analyses. Taking any medication(s) for a medical condition was examined independently of the medical condition as a medication yes/no variable. We also investigated nitroglycerine use, alone.

Height was measured using a Handi-stat set to the nearest 0.1 cm, and weight was determined using an electronic scale. Body mass index (BMI) was calculated as weight(kg)/height(m^2^). Systolic and diastolic blood pressure (DBP) were obtained sitting with an automated blood pressure machine and averaged over three measurements. Hypertension was defined as a mean SBP ≥140 mmHg, or a mean DBP ≥90 mmHg, or taking antihypertensive medication, or self-report of a doctor’s diagnosis of hypertension.

Peripheral blood was collected after at least 8 hours of fasting, and serum was retained from the samples for measurement of fasting glucose, hemoglobin A1c (HbA1c), low-density lipoprotein cholesterol (LDL), high-density lipoprotein cholesterol (HDL), triglycerides, and creatinine. Glucose was measured using the Roche hexokinase method (Roche Diagnostics, Indianapolis, IN, USA) on a Roche Modular P Chemistry Analyzer. HbA1c was measured in EDTA whole blood on the Tosoh HPLC Glycohemoglobin Analyzer (Tosoh Medics, Inc., San Francisco, CA, USA) using an automated high performance liquid chromatography method. The laboratory CV is 1.6% and 1.4–1.9% for fasting serum glucose and HbA1c, respectively. Diabetes was defined as fasting serum glucose ≥126 mg/dL, or HbA1c ≥6.5, or taking diabetes medication, or self-reporting of a doctor’s diagnosis of diabetes mellitus. Fasting serum cholesterols were measured as follows: total cholesterol was measured using a cholesterol oxidase method (Roche Diagnostics, Indianapolis, IN, USA). HDL-cholesterol was measured directly in serum using the Roche HDL-Cholesterol 3rd generation direct method (Roche Diagnostics, Indianapolis, IN, USA). Triglycerides were measured in serum using Triglyceride GB reagent (Roche Diagnostics, Indianapolis, IN, USA). LDL-cholesterol was then calculated by the Friedewald equation [36] using the measured results for total cholesterol, HDL-cholesterol, and triglycerides. The laboratory CVs are 1.6%, 2.9%, and 4.0% for total cholesterol, HDL-cholesterol and triglycerides, respectively. Dyslipidemia was defined as LDL ≥100 mg/dL, or HDL ≤40 mg/dL, or triglycerides ≥150 mg/dL, or taking a lipid-lowering medication, such as a statin. Serum creatinine was measured using the Roche enzymatic method (Roche Diagnostics, Indianapolis, IN, USA). The laboratory CV is 2.3% for creatinine.

### Genotyping, linkage markers, and imputation

The Center for Inherited Disease Research assayed all LLFS subjects who provided DNA samples and consent using the Illumina Human Omni 2.5 v1 chip. Haplotypes were generated from these data and used to estimate multipoint identity-by-descent estimates in Loki [37] with a sex-specific map for use in linkage analysis. Details of the genotyping, quality control, haplotype generation, and imputation in LLFS have been published [38]. Briefly, genotypes were called using Bead Studio, and data were phased using Eagle software (v2.4) [39]. Individuals with a ≥2% missing rate were dropped. In addition, 1.4 million genotyped SNPs passed quality control protocol including: a call rate of greater than 0.95, a minor allele frequency of >0.1%, and no deviation from Hardy-Weinberg Equilibrium (p >1E-06). Imputation was completed on the Michigan Imputation server [40] using the TOPMed Reference Panel Version 5b [41] and required an imputation quality threshold of r2 >0.3 using minimac4 (v1.3.3) [42].

Loki [43] was run to assess Mendelian errors on autosomal chromosomes, and familial relationships were verified based on identity-by-state. In each half cM interval, the first five SNPs were haplotyped with ZAPLO [44]. These SNP haplotype bins were used in Loki [43] to estimate multipoint IBDs (mIBDs) at 1cM intervals, and there were 3 distinct sets of IBDs created using all and two alternating sets of bins. The current analyses included all three sets of IBDs to examine linkage peaks and control for poorly estimated markers via evaluation across sets.

### Statistical analysis

ABI was log-transformed to approximate normality due to skewness. To identify important predictors of ABI and PAD, we used stepwise linear (ABI) and logistic (PAD) regression models to identify significant independent correlates in the LLFS. All stepwise models included adjustment for age, sex, and field centers, and the alpha to enter was set at 0.2 and to stay was 0.05. To minimize the chance for overfitting the models, variables reflecting prevalent medical conditions were not included in the selection procedures, but the factors that defined their prevalence, including medication usage, were (e.g., SBP, DBP, antihypertensive medications were added to model selection, but hypertension was not). To investigate the proportion of ABI and PAD determined by genetic variation, we estimated residual genetic heritability using the maximum likelihood methods as implemented in SOLAR, which accounts for family relatedness [45]. All heritability analyses were adjusted for age, sex, field centers, and significant independent predictors identified in the model selection procedures.

Genetic association analyses were completed using R version 4.2.0 (R Core Team, Vienna, Austria) with GENESIS version 2.26.0 [46]. Each GWAS included only SNPs with a minor allele frequency >0.01 after imputation (quality r2 >0.3), resulting in approximately 8 million SNPs. The base model was adjusted for age, sex, field centers, significant PCs of population substructure, and familial relatedness. Significant PCs were determined through a stepwise linear regression model and familial relatedness was accounted for using the GENESIS package [46]. Full adjustment additionally included the identified significant independent correlates from above. All models used a *P*-value < 5 × 10^−8^ to indicate significance. Additionally, the fully adjusted GWAS of ABI and PAD were completed and stratified by proband and offspring generation, including all spousal controls, to identify any genetic effects that may vary in expression by age. Due to low case numbers in the offspring, PAD stratified analyses were only conducted in the proband generation. Manhattan plots were generated for each analysis and LocusZoom plots [47] were created for loci with significant findings.

For genomewide linkage analyses, the significance of a theoretical quantitative trait locus (QTL) was tested with a likelihood ratio test at 1 centi-Morgan (cM) intervals across each autosomal chromosome in SOLAR adjusted for age, sex, field centers in the base model. Fully adjusted models also included the identified significant independent correlates from above. All three sets of generated mIBD files were analyzed to ensure results were similar and any significant findings were not due to sporadic errors in mIBD estimation or genotyping. Logarithm of the odds (LOD) scores, computed as the log_10_ of the likelihood ratio, were used to assess the significance of the test with LOD ≥3.0 and 2.5, indicating genomewide significant and suggestive evidence, respectively [48, 49]. Similar to association analyses, linkage analyses were also conducted stratified by generation to assess variation in results by age.

## Supplementary Materials

Supplementary Figures

Supplementary Table 1
