# Supplementary material for: Epidemiology and genetic determination of measures of peripheral vascular health in the Long Life Family Study
Source: Aging (Albany NY). 2025 Feb 25;17(2):464–81. doi: 10.18632/aging.206204 (PMC11892930; doi:10.18632/aging.206204)
Supplement: Supplementary Figures [file aging-17-206204-s001.pdf]

SUPPLEMENTARY FIGURES

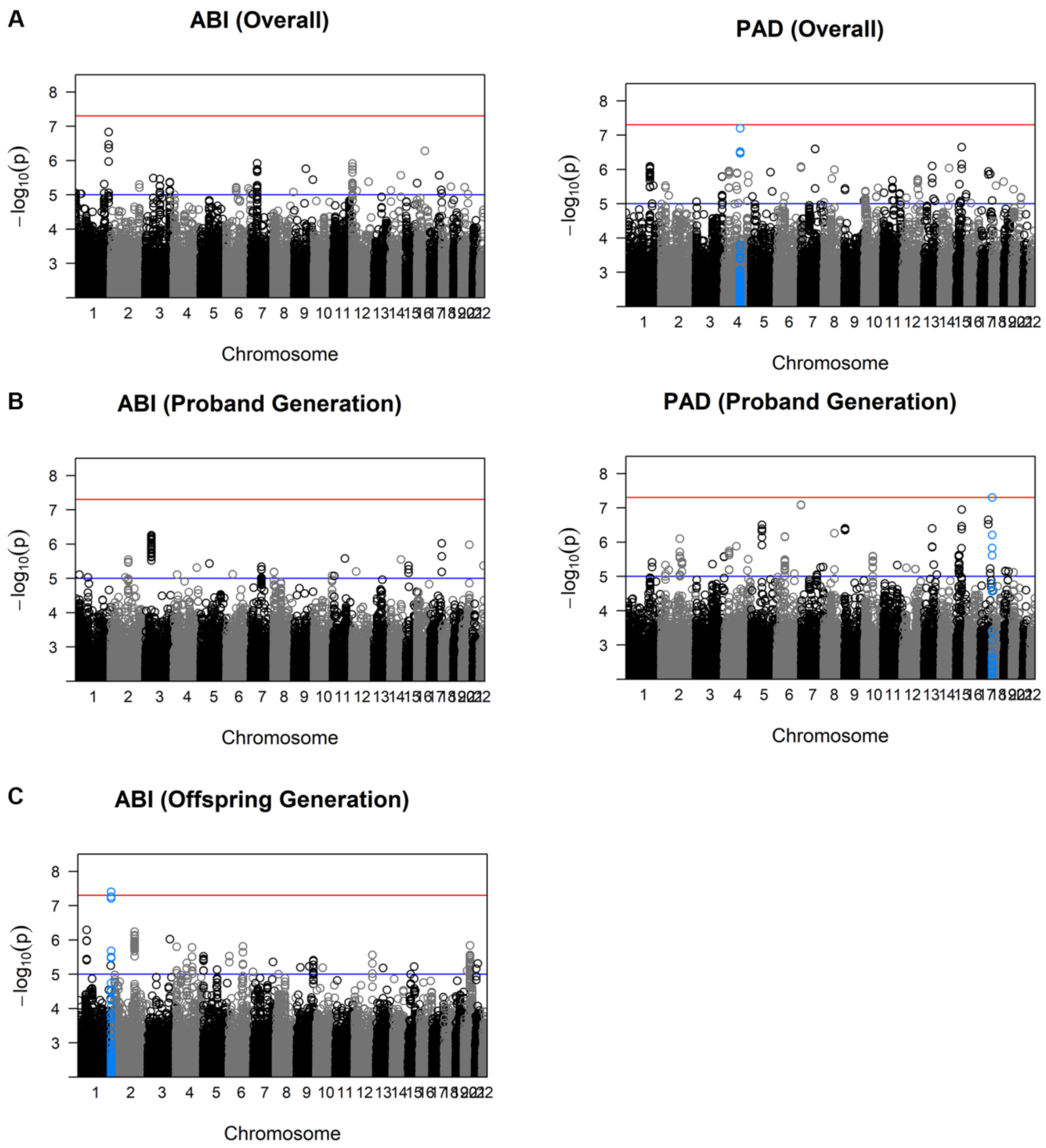

Supplementary Figure 1. GWAS results for ABI and PAD overall and stratified by generation. Shown are fully adjusted genomewide association study results for ABI (left panels) and PAD (right panels) overall (**A**), as well as, stratified by generation ((**B**) Proband, (**C**) Offspring). Plots include lines representing suggestive (blue) and significant (red) *p*-values. Due to very low prevalence of PAD in the offspring generation, a GWAS for PAD in the offspring generation was not performed. To further highlight significant results in the plot, all SNPs located within 500 kb upstream and downstream of significant lead SNPs are highlighted in blue.

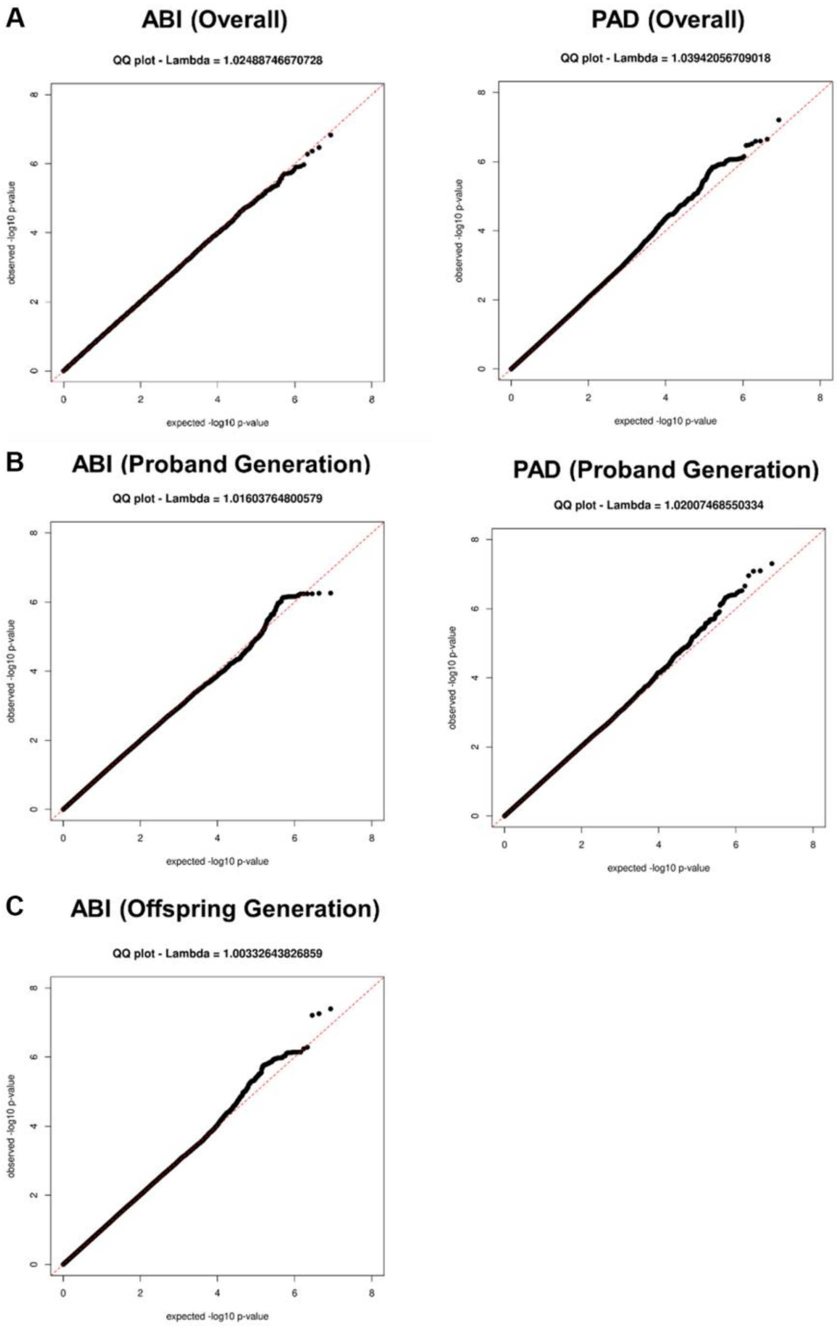

Supplementary Figure 2. Q-Q plots for ABI and PAD GWAS analyses in the overall and generation-stratified groups. Shown are Q-Q plots for genomewide association tests of ABI (left panels) and PAD (right panels) overall (**A**), as well as, stratified by generation ((**B**) Proband, (**C**) Offspring). Lambda values are reported at the top of each panel, and non are >1.04 suggesting no genomic inflation.

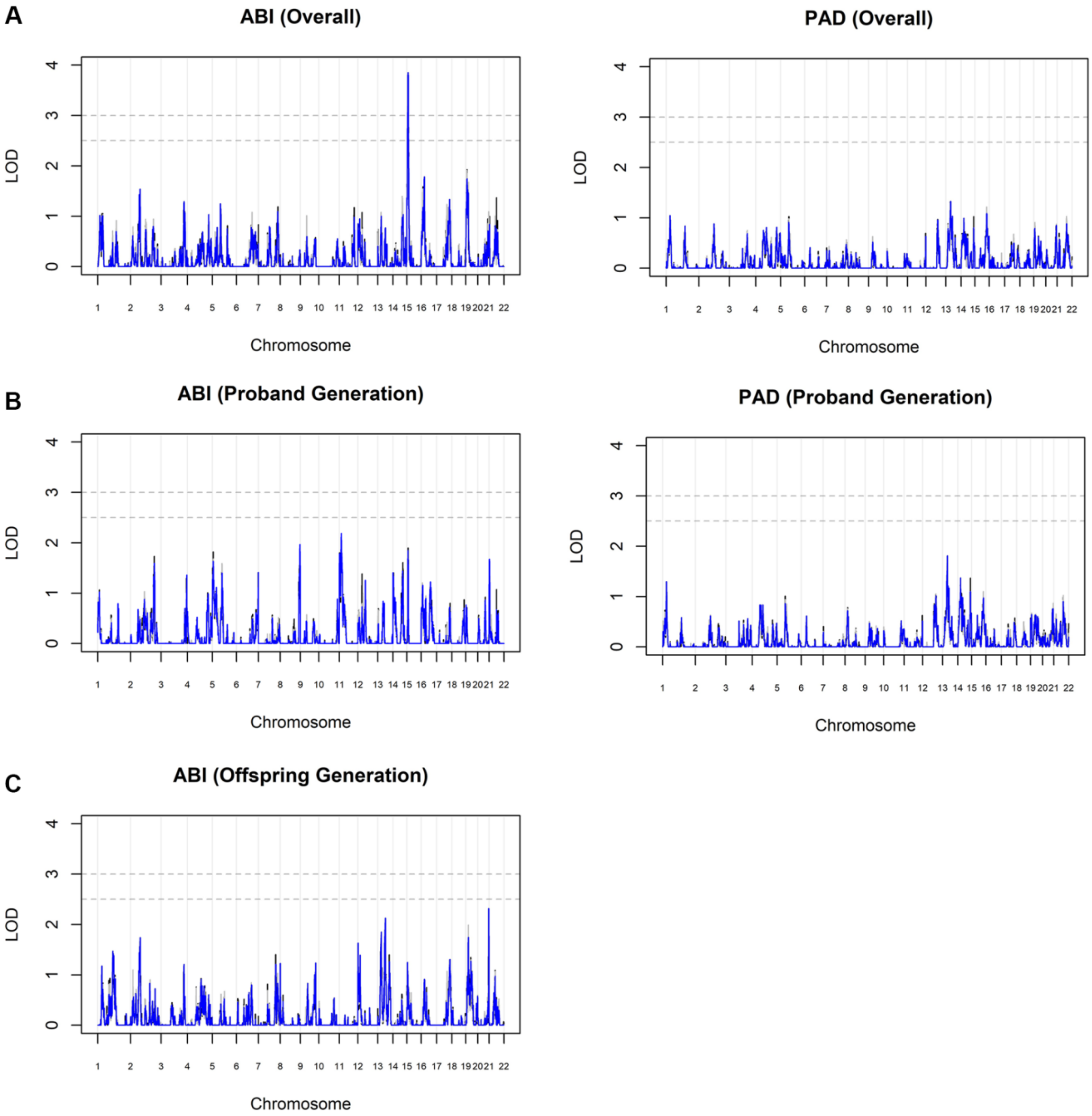

Supplementary Figure 3. Genomewide linkage results for ABI and PAD overall and stratified by generation. Shown are fully adjusted genomewide linkage results for ABI (left panels) and PAD (right panels) overall (**A**), as well as, stratified by generation ((**B**) Proband, (**C**) Offspring). Plots include dashed lines representing suggestive (LOD = 2.5) and significant (LOD = 3.0) LOD scores. Due to very low prevalence of PAD in the offspring generation, genomewide linkage for PAD in the offspring generation was not performed.
