# Supplementary material for: Epidemiology and genetic determination of measures of peripheral vascular health in the Long Life Family Study
Source: Aging (Albany NY). 2025 Feb 25;17(2):464–81. doi: 10.18632/aging.206204 (PMC11892930; doi:10.18632/aging.206204)
Supplement: Supplementary Table 1 [file aging-17-206204-s002.pdf]

SUPPLEMENTARY TABLE

Supplementary Table 1. Distribution of peripheral vascular health measures in the LLFS.

	Overall (N = 3006)	Proband generation (N = 1090)	Offspring (N = 1554)	Spousal controls (N = 362)	P-value for offspring v. spousal controls[a]
Median ABI	1.16	1.10	1.19	1.20	0.538
PAD (%)	7.4	18.2	1.0	1.9	0.541

[a]Adjusted for age, sex, and field centers. Mean age (range) of offspring was 60.1 (30–87) years and spousal controls was 60.9 (24–83) years (P for difference = 0.07). Offspring were 60.1% female, while spousal controls were 48.3% (P for difference < 0.0001).
